# Anterior-posterior gene expression differences in three Lake Malawi cichlid fishes with variation in body stripe orientation

**DOI:** 10.7717/peerj.4080

**Published:** 2017-11-17

**Authors:** Ehsan Pashay Ahi, Kristina M. Sefc

**Affiliations:** Institute of Zoology, Universitätsplatz 2, Universität Graz, Graz, Austria

**Keywords:** Cichlids, Gene expression, Pigmentation, Oblique stripe, Lake malawi, Melanophores, Iridophores

## Abstract

Morphological differentiation among closely related species provides opportunities to study mechanisms shaping natural phenotypic variation. Here, we address variation in the orientation of melanin-colored body stripes in three cichlid species of the tribe Haplochromini. * Melanochromis auratus* displays a common pattern of dark, straight horizontal body stripes, whereas in * Aristochromis christyi* and *Buccochromis rhoadesii*, oblique stripes extend from the anterior dorsal to the posterior mid-lateral trunk. We first validated a stably reference gene, and then, investigated the chromatophore distribution in the skin by assessing the expression levels of the iridophore and melanophore marker genes, *ltk* and *slc24a5*, respectively, as well as *pmel*, a melanophore pigmentation marker gene. We found anterior-posterior differences in the expression levels of the three genes in the oblique-striped species. The higher anterior expression of *ltk*, indicates increased iridophore density in the anterior region, i.e., uneven horizontal distribution of iridophores, which coincides with the anterior dorsalization of melanophore stripe in these species. The obliqueness of the horizontal body stripes might be a result of distinct migratory or patterning abilities of melanophores in anterior and posterior stripe regions which could be reflected by variation in the expression of genes involved in melanophore patterning. To address this, we investigated anterior-posterior expression levels of a primary set of candidate target genes with known functions in melanophore migration and stripe patterning in the adult zebrafish, and their related gene regulatory network. Among these genes, those with differences in anterior-posterior expression showed only species-specific differential expression, e.g., *sdf1a*, *col14a1a*, *ifitm5*, and *agpat3*, with the exception of *fbxw4/hagoromo* (differentially expressed in an oblique-and the straight-striped species). In summary, distinct anterior-posterior gradients in iridophore density found to be more similar characteristic between the two oblique-striped species. Furthermore, the species-specific differential expression of genes involved in stripe patterning might also implicate distinct molecular processes underlying the obliqueness of body stripe in two closely related cichlid species.

## Introduction

Fish are renowned for their diverse color patterns. These patterns include horizontal stripes, vertical bars, as well as clearly defined or gradually shading patches of color (Parichy, 2003; Kelsh, 2004). On a cellular basis, patterns are produced by variation in the concentration and distribution of chromatophores and in the content and distribution of pigments or refractors within these cells (Fujii, 2000; Kelsh et al., 2009; Kobayashi et al., 2012). Fish melanophores synthesize only one type of melanin, the dark eumelanin. The yellow and red hues of xanthophores and erythrophores are produced by pteridine pigments, which are synthesized *de novo*, and by carotenoids obtained from the diet. Blue, green, white and metallic hues are produced by the reflection of light from purine crystals arranged in iridophores and leucophores (Kelsh, 2004; Braasch, Volff & Schartl, 2008; Mills & Patterson, 2009). The molecular mechanisms contributing to color pattern morphogenesis have been studied extensively in the zebrafish, whose adult pattern consists of alternating dark and light stripes along the body and fins (Parichy, 2003; Singh & Nüsslein-Volhard, 2015). The formation of the dark horizontal stripes is accomplished by the migration, differentiation and death of melanophores and their precursors. Intriguingly, the adult stripe pattern is determined by interactions between chromatophores rather than a predetermined patterning mechanism (Nakamasu et al., 2009; Frohnhöfer et al., 2013) and melanophores retain migration and patterning abilities during adulthood (Takahashi & Kondo, 2008). Co-attraction and contact inhibition between chromatophores of the same kind (homotypic interactions) are necessary for their dispersal in skin (Walderich et al., 2016). Furthermore, heterotypic interactions between chromatophores across both long and short distances are required for the formation and maintenance of stripe patterns (Nakamasu et al., 2009). For example, short distance inhibitory interactions with iridophores do not allow melanophores to settle within the light regions of zebrafish skin, but iridophores promote melanophore aggregation nearby through long distance attraction (Patterson & Parichy, 2013; Frohnhöfer et al., 2013). Also, melanophores tend to migrate to iridophore-free sites (Patterson & Parichy, 2013) and when encountering an expanding region of dense iridophores, their shape changes and they eventually disappear (Nüsslein-Volhard & Singh, 2017). At the molecular level, a number of genes involved in adult stripe formation and/or maintenance have already been identified in the zebrafish (Singh & Nüsslein-Volhard, 2015) and can readily be tested as candidates contributing to the natural variation of color patterns across different groups of fishes (Ahi & Sefc, 2017).

**Figure 1:**
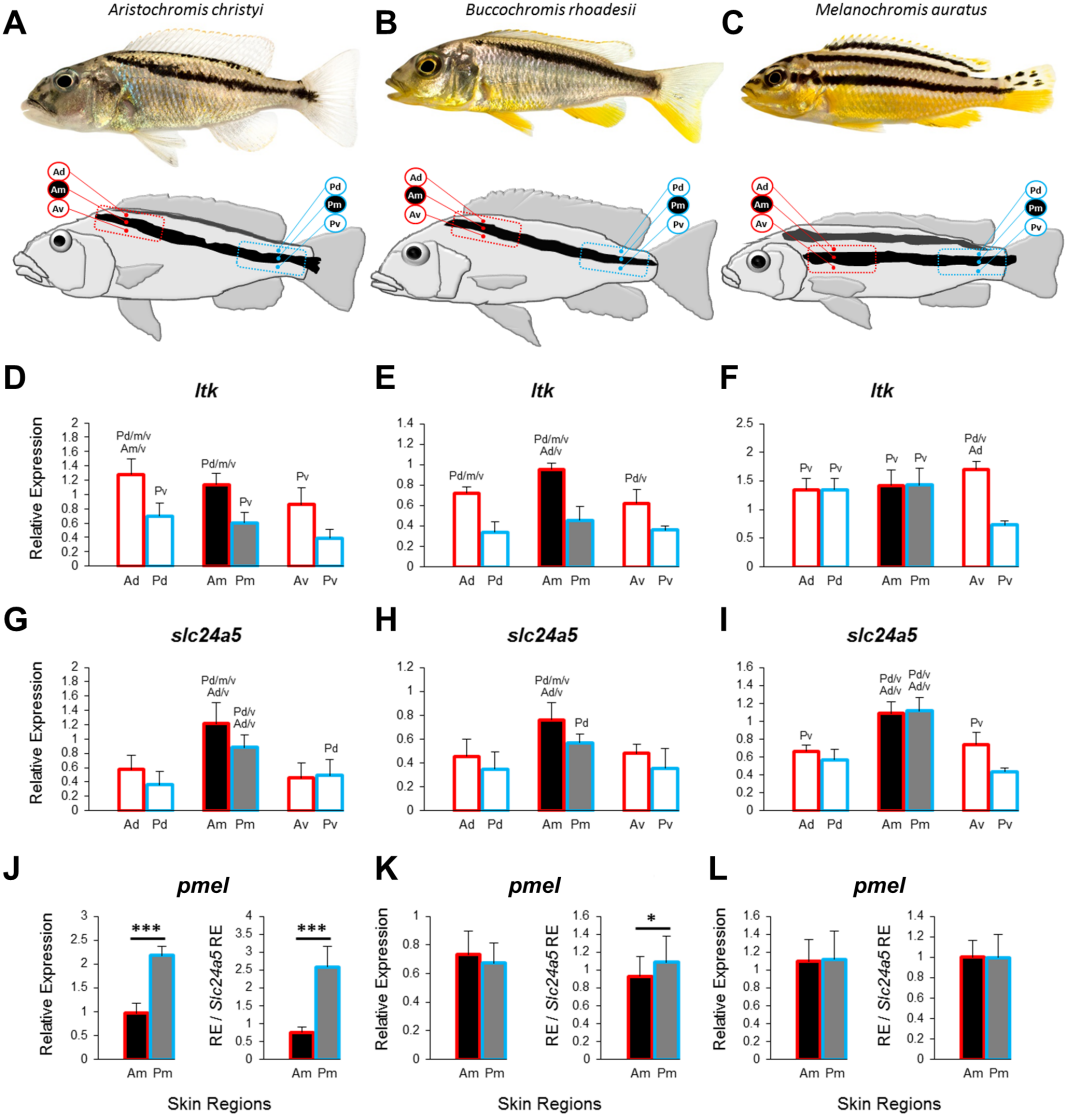
Three Lake Malawi cichlid species and expression of chromatophore marker genes in sampled skin regions. (A–C) Young adult females of three Lake Malawi cichlid species display distinct body stripe patterns. The red and blue boxes mark the anterior and posterior location, respectively, of the sampled skin, which was further dissected into dorsal, middle, and ventral tissue samples, relative to the dark stripe, as indicated by the skin region labels (Ad, anterior dorsal; Am, anterior middle; Av, anterior ventral; Pd, posterior dorsal; Pm, posterior middle; Pv, posterior ventral). (D–I) The expression levels of *ltk* and *slc24a5* in all the skin regions depicted above. (J–L) The expression levels of *pmel* in the stripe region (Am, Pm). Division of relative *pmel* expression levels by the relative expression levels of the melanophore marker gene *slc24a5* corrects for differences in melanophore numbers among Am and Pm stripe regions. Statistical comparisons were conducted among the skin regions and significant differences between them are indicated by the letter codes of the skin regions with significantly lower expression above bars (*P* < 0.05). Error bars represent standard deviations calculated from five biological replicates and RE indicates relative expression.

Horizontal stripes—as in zebrafish—and vertical bars are frequent motifs of melanin-based color patterns across fish species. Both stripes and bars are also found within the highly diverse and species-rich cichlid fish family (Maan & Sefc, 2013), but additionally, variations of these basic patterns exist. In the present study, we focus on a particular modification of the horizontal stripe pattern, namely the display of oblique melanin-colored stripes, which extend at an angle from an anterior dorsal position behind the head to a mid-lateral position at the end of the caudal peduncle, in *Aristochromis christyi* and *Buccochromis rhoadesii* from Lake Malawi, East Africa (Figs. 1A, 1B). While dominant adult males lose or reduce their stripe pattern in favor of uniform coloration, females of these species retain the distinct stripes into adulthood. In females of both species, the dark stripes are surrounded (ventrally and dorsally) by white, presumably iridophore-rich areas. The third species addressed in our study, *Melanochromis auratus*, exhibits horizontal stripes (Fig. 1C), and serves as a straight-striped comparison to the two oblique-striped species. In the cichlid tribe Haplochromini, which radiated into hundreds of endemic species in Lake Malawi within the last 700–800 ky (Malinsky et al., 2017), *M. auritus* is a member of the rock-dwelling ‘mbuna’ clade, while *A. christyi* and *B. rhoadesii* belong to a clade of sand-dwellers (Malinsky et al., 2017; Hulsey et al., 2017).

The oblique orientation of the dark stripes could be driven by cellular and molecular mechanisms such as presence of anterior-posteriorly distinct interactions between chromatophores, since iridophores determine the organization of melanophores in the skin through short and long distance interactions (Patterson & Parichy, 2013; Frohnhöfer et al., 2013), and/or by differences in the intrinsic migration abilities of melanophore populations along the anterior-posterior axis. An initial step towards our understanding of such mechanism(s) is the characterization of iridophores/melanophores along the horizontal stripe including their distribution in the skin, morphology and migratory abilities. Here we used gene expression analysis to address some of these properties in the three cichlid species. To characterize the distribution of melanophores/iridophores along the body axis we examined the expression of the chromatophore marker genes, *ltk* and *slc24a5*, in the stripes and their adjacent skin regions (Fadeev et al., 2016; Lamason et al., 2005). In zebrafish, melanophore stripe formation also depends on interactions with xanthophores (Mahalwar et al., 2014). Since we found no yellow or red coloration in the area immediately surrounding the dark stripe in the here studied cichlids, we concentrate on iridophores only. Furthermore, to examine possible differences in pigmentation related properties of melanophores along the stripes, we tested the expression of *pmel*, a gene determining the shape and melanin localization of melanophores (Schonthaler et al., 2005), together with members of its predicted gene regulatory network. Finally, we also compared the expression of 11 candidate genes associated with melanophore migration and stripe formation in the zebrafish (see references in Table 1) between anterior and posterior sections of the stripes. Based on the signals obtained from this primary set of candidates, we extended our investigation to co-expressed genes and to predicted upstream regulators. Candidate genes differing in their anterior-posterior expression patterns between the oblique-striped and the straight-striped species potentially contribute to stripe orientation.

**Table 1:** Selected candidate genes involved in melanophore stripe and/or adult pigmentation in fish.

Gene	Related function	Adult pigmentation^a^	Stripe formation	References
*bnc2*	A Znf protein controlling melanophore survival expressed by hypodermal cells near to chromatophores	+	+	Lang et al. (2009)
*fms/csf1r*	A PDGF receptor essential for directional melanophore migration and survival with axially distinct effects	+	+	Parichy et al. (2000) and Parichy & Turner (2003)
*ednrb1*	A tyrosine kinase receptor controlling the normal patterning of melanocyte death and migration	+	+	Parichy et al. (2000)
*erbb3b*	A EGF receptor promoting formation of melanophores and adult pigment pattern with axially distinct effects	+	+	Budi, Patterson & Parichy (2008)
*fbxw4/hagoromo*	An F-box protein required for melanophore stripe organization with axially distinct effects	+	+	Kawakami et al. (2000)
*igsf11*	An immunoglobulin member mediating adhesive interactions, migration and survival of melanophores	+	+	Eom et al. (2012)
*kita*	A tyrosine kinase receptor required for melanophore survival and formation during both early development and adult pigmentation	+	+	Parichy et al. (1999), Mills, Nuckels & Parichy (2007) and Dooley et al. (2013)
*mitfa*	A transcription factor involved in diverse aspects of melanophore differentiation	+	+	Lister et al. (1999) and Johnson, Nguyen & Lister (2011)
*mmp2, mmp14*	Members of ECM remodelling enzymes contributing to tissue invasion ability of melanophores	?	+	Ellis & Crawford (2016)
*sdf1a*	A chemokine essential for the lateral stripe patterning through controlling the invasion of melanophores	?	+	Svetic et al. (2007)

**Notes.**

a A role in adult pigmentation mainly indicates the requirement of gene function for survival of different chromatophore lineages or/and pigment formation in adult zebrafish.

## Methods

### Sampling of fish skin

Skin tissue samples were obtained from 15 captive bred young adult females, 5 individuals per species, of *Aristochromis christyi*, *Buccochromis rhoadesii* and *Melanochromis auratus*. The total length of the sampled individuals was 6–7 cm for *A. christyi* and *B. rhoadesii*, and around 5 cm for *M. auratus*. The fish were housed in a single large aquarium and fed on similar diets for one month. To acquire skin tissues, the fishes were euthanized in water with 0.1 gram MS-222/litre, and after quick and careful removal of the scales covering the mid-lateral dark stripe and the area dorsal and ventral around it, the skin was dissected from an anterior and an posterior region (tissue samples designated A and P, respectively) along the stripe, as shown by the red and blue squares on the fish drawings in Figs. 1A–1C. We noticed that pigmentation of the scales contributed to the color pattern, but nonetheless removed scales in order to avoid variation in skin and scale content among tissue samples and to circumvent potential complications due to morphological differences among scales along anterior-posterior body axis (Ibanez, Cowx & O’Higgins, 2009). Each A or P tissue was cut into 3 parts, the white-reflecting one dorsal of the dark stripe (designated Ad and Pd, with ‘d’ for dorsal), one covering the dark stripe (Am and Pm, with ‘m’ for middle), and the white-reflecting one ventral to the dark stripe (Av and Pv, with ‘v’ for ventral, Figs. 1A–1C). Tissue samples were stored in RNAlater (Thermo Fisher Scientific) at −20 °C. The research was approved by the Federal Ministry of Science, Research and Economy of Austria (approval number: BMWFW-66.007/0004-WF/V/3b/2016).

### RNA isolation and cDNA synthesis

Corresponding tissue samples from each fish were treated as biological replicates (*n* = 5 replicates per species) and placed into tubes with TRIzol Reagent (Sigma) and 1.4 mm ceramic spheres. The samples were homogenized by FastPrep-24 tissue disruptor (MP Biomedicals Europe). RNA was isolated following the manufacturer’s Trizol protocol and dissolved in 30 µl Nuclease-free water. The genomic DNA was removed from RNA samples using DNase I (NEB) and RNA quantity was estimated by Nanophotometer (Pearl; Implen, Munich, Germany). The RNA quality was assessed in a R6K ScreenTape System on an Agilent 2200 TapeStation (Agilent Technologies, Santa Clara, CA, USA) and samples with RIN number above 6 were kept for next step. cDNA was synthesized from ∼400 ng of RNA through the High Capacity cDNA Reverse Transcription kit (Applied Biosystems, Foster City, CA, USA). Negative controls with no reverse transcriptase were included in order to verify the absence of DNA contamination.

### Gene selection, primer design and real-time qPCR

To identify stably expressed reference genes for further analysis, we screened 6 genes which are known to be expressed in different tissues and often used as reference genes for qPCR analysis in fishes (Table S1) (Yang et al., 2013; Ahi et al., 2013; Pashay Ahi et al., 2016; Ahi & Sefc, 2017). Expression levels of the melanosome marker gene *slc24a5*, which encodes an intracellular membrane cation exchanger predominantly present in melanosomes (Lamason et al., 2005), and the iridophore lineage specific marker *ltk,* which encodes a tyrosine kinase receptor required for iridophore differentiation and stripe formation in adult zebrafish (Lopes et al., 2008), were quantified as proxies for the densities of the two chromatophore types. The expression levels of both genes have been shown to be tightly associated with numbers of iridophores and melanophores (Fadeev et al., 2016; Ahi & Sefc, 2017; Lamason et al., 2005). To examine potential differences in structural properites of melanophores along the dark stripes, we quantified the expression of *pmel/silv*, which encodes a melanophore-specific transmembrane glycoprotein with an essential role in the organization of premelanosomes, melanin localization and determination of melanophore shape (Schonthaler et al., 2005).

To examine the genetic control of stripe formation and orientation, we selected a primary set of 11 candidate target genes known to be involved in adult pigmentation and stripe formation in zebrafish (Table 1). We extended our expression analyses to genes co-expressed with genes that showed expression differences between Am vs Pm (the stripe regions) using COXPRESdb (http://coxpresdb.jp/) version 6.0 (Obayashi & Kinoshita, 2011). The database contains gene co-expression networks identified across model vertebrates, including zebrafish. To acquire coexpressed candidate genes with a high level of fidelity, we limited the genes by setting the Supportability to 1 value (Obayashi & Kinoshita, 2011) (Table S2). The top eight genes co-expressed with each candidate were tested in a first step, and after identification of a gene showing similar expression difference, the top 5 genes co-expressed with both genes were tested in a second step (this stepwise approach is described by Ahi et al., 2015).

To predict the potential upstream transcription factors, we conducted motif enrichment analysis in the region encompassing 2 kb upstream of the transcription starting sites of interesting genes emerging from the previous steps, using the annotated genome of the Nile tilapia (Flicek et al., 2013) as well as two algorithms: MEME (Bailey et al., 2009) and XXmotif (Luehr, Hartmann & Söding, 2012). We proceeded with the motifs that were enriched in the tested promoters and screened for potential transcription factor (TF) binding sites through STAMP (Mahony & Benos, 2007) and the motif position weight matrices (PWMs) obtained from the TRANSFAC database (Matys et al., 2003) (Table S3).

The qPCR primers were designed based on conserved coding sequences of four East African cichlids with annotated transcriptome data (Brawand et al., 2014). Among these four species, *Maylandia zebra* is most closely related to our study species, as it also belongs to the Haplochromini clade of Lake Malawi. *Pundamilia nyererei* is a member of the same tribe, Haplochromini, but belongs to another clade that radiated in the Lake Victoria region. Coding sequences of the genes targeted in this study are 99–100% identical between *M. zebra* and *P. nyererei*. Additionally, *Neolamprologus brichardi* (Lamprologini) and *Oreochromis niloticus* (the Nile tilapia, Oreochromini) represent more distant relatives of the target species. Primers were designed on sequences that were conserved across the four species to ensure their match to the targeted Haplochromini species of Lake Malawi. In addition, primers were designed with melting temperatures of 2−3 °C degree higher than the temperature of qPCR extension stage (see below) which could allow a single mismatch per primer to still result in successful amplification (Table S1). The primers were positioned across exon junctions determined in the Nile tilapia annotated genome in the Ensembl (http://www.ensembl.org/Oreochromis_niloticus). Primer Express 3.0 software (Applied Biosystems, Foster City, CA, USA) and OligoAnalyzer 3.1 (Integrated DNA Technology, Coralville, IA, USA) were used for primer design and checking structural properties (e.g., self-annealing and hetero-dimers) (Table S1).

qPCR was implemented on an ABI 7500 real-time PCR System (Applied Biosystems) using Maxima SYBR Green/ROX qPCR Master Mix (2X) following the manufacturer’s instruction (Thermo Fisher Scientific, St Leon-Rot, Germany) and the experimental set-up was conducted based on the preferred sample maximization approach (Hellemans et al., 2007). The qPCR amplification was set with a 2 min hold at 50 °C, a 10 min hot start at 95 °C, and 40 cycles of 15 s denaturation at 95 °C and 1 min extension at 59 °C. A dissociation step (60 °C–95 °C) was conducted at the end of the amplification to verify a single, specific product for each primer pair (Table S1). Primer efficiency values (E) were estimated by LinRegPCR v11.0 software (http://LinRegPCR.nl) (Ramakers et al., 2003) and primer-pairs with E less than 0.9 were discarded and new primers designed (Table S1).

### Data analysis

To measure the stability of the reference gene candidates, three algorithms were used; BestKeeper (Pfaffl et al., 2004), NormFinder (Andersen, Jensen & Ørntoft, 2004) and geNorm (Vandesompele et al., 2002). BestKeeper determines the stability of reference genes through a correlation calculation index (r). GeNorm estimates mean pairwise variation between each gene and other candidates (*M* value) and NormFinder identifies the most stable genes (lowest expression stability values) through analysis of inter- and intra-group variation in expression levels (Ahi et al., 2013; Pashay Ahi et al., 2016).

The Cq values of the top-ranked reference gene (across the three species) was used as Cq_reference_ and the difference between Cq values (ΔCq) of the target genes and the reference gene was calculated for each target gene; ΔCq_target_ = Cq_target_ − Cq_reference_. To calculate a ΔΔCq value, samples were normalized to the ΔCq value of a calibrator sample (ΔCq_target_ − ΔCq_calibrator_). To this aim, one arbitrarily chosen biological replicate of Am (see Figs. 1A–1C) was used to calibrate samples within each species. Relative expression quantities (RQ) were calculated based on the expression level of the calibrator sample (2^−ΔΔCq^) (Pfaffl, 2001). For each target gene, differences in gene expression levels between the corresponding anterior and posterior tissue samples were tested by paired *t*-tests on log-transformed RQ data.

## Results

### Validation of reference genes

Gene expression analyses by qPCR rely on the validation of stably expressed reference genes (Kubista et al., 2006), and depending on species, tissue and experimental conditions, the expression stability of reference genes can be variable (Ahi et al., 2013). To identify suitable reference genes for our study, we examined the expression of six reference gene candidates in cDNA of the tissue samples from the six skin parts for each species. For two of the species, *A. christyi* and *B. rhoadesii*, the expression levels of these candidate genes from highest to lowest (lowest to highest Cq) were *gapdh* > *actb1* > *rps11* > *rps18* > *hsp90a* > *hprt1*, whereas in *M. auratus actb1* had higher expression than *gapdh* (*actb1* > *gapdh*) (Table S4). Based on the three algorithms implemented in BestKeeper, geNorm and NormFinder, *rps11* consistently ranked among the top two genes in terms of expression stability in the three species (Table 2). Therefore, expression of *rps11* was used to normalize target gene expression for quantitative comparisons between skin regions.

**Table 2:** Ranking of candidate reference genes using BestKeeper, geNorm and NormFinder in skin samples of three cichlid species.

	BestKeeper		geNorm		NormFinder	
	Ranking	r	Ranking	M	Ranking	SV
*Aristochromis christyi*	*rps11*	0.968	*rps11*	0.539	*rps11*	0.143
	*hsp90a*	0.955	*rps18*	0.544	*rps18*	0.202
	*rps18*	0.951	*actb1*	0.583	*actb1*	0.310
	*hprt1*	0.917	*hprt1*	0.654	*hprt1*	0.406
	*actb1*	0.913	*hsp90a*	0.674	*hsp90a*	0.475
	*gapdh*	0.332	*gapdh*	1.277	*gapdh*	0.986
*Buccochromis rhoadesii*	*rps11*	0.940	*rps11*	0.609	*rps11*	0.156
	*rps18*	0.876	*rps18*	0.614	*rps18*	0.193
	*actb1*	0.870	*actb1*	0.628	*actb1*	0.275
	*hprt1*	0.844	*hsp90a*	0.831	*hprt1*	0.543
	*hsp90a*	0.810	*hprt1*	0.955	*hsp90a*	0.555
	*gapdh*	0.505	*gapdh*	1.315	*gapdh*	0.881
*Melanochromis auratus*	*actb1*	0.925	*rps11*	0.865	*actb1*	0.182
	*rps11*	0.874	*actb1*	0.897	*rps11*	0.203
	*hsp90a*	0.782	*rps18*	0.912	*rps18*	0.487
	*rps18*	0.749	*hsp90a*	1.037	*hsp90a*	0.584
	*gapdh*	0.544	*hprt1*	1.258	*hprt1*	0.865
	*hprt1*	0.535	*gapdh*	2.180	*gapdh*	1.497

**Notes.**

Abbreviations r Pearson product-moment correlation coefficient SV stability value M M value of stability

### Expression of iridophore and melanophore marker genes

To investigate the distribution of chromatophores, we quantified the expression of the iridophore lineage specific marker *ltk* (Lopes et al., 2008) and the melanosome marker *slc24a5* (Lamason et al., 2005) in the dark stripe as well as in the dorsally and ventrally adjacent light-coloured areas. In the two species with oblique stripes, *A. christyi* and *B. rhoadesii*, expression of *ltk* was higher in the anterior skin samples compared to their posterior counterparts (Figs. 1D, 1E), indicative of an anterior-posterior decline of iridophore density. In the straight-striped *M. auratus*, an anterior-posterior difference in *ltk* expression was detected only ventral of the dark stripe (Fig. 1F). Notably, *ltk* was expressed at similar levels within and adjacent to the stripe regions of all three species. The high expression level of *ltk* in the dark stripe indicates the presence of iridophores in these regions as well, but possibly in different layers than the melanophores, as observed in zebrafish (Hirata et al., 2003).

As expected, expression of the melanosome marker, *slc24a5*, was generally higher within the stripe than in the adjacent light-colored tissue (Figs. 1G–1I). Although the contrast between the dark stripe and the adjacent areas is strong in the live fish (Figs. 1A–1C), the *slc24a5* expression differences were not large, and not even significant in the posterior region of *B. rhoadesii*. Melanophores were observed in the light skin next to the stripes (EP Ahi, pers. obs., 2017), but most of the discrepancy between live color contrast and *slc24a5* expression differences in the skin samples was probably due to the removal of scales, which were strongly pigmented along the stripe, in the course of sample preparation. In the two oblique-stripe species, *slc24a5* expression was higher in the anterior than in the posterior stripe sample, indicating a gradient in melanophore density. Finally, we examined the expression of *pmel*, a gene determining melanophore shape and the intra-organelle localization of melanin, along the stripe. In order to investigate *pmel* expression as a property of the melanophores, i.e., independent of melanophore density in the investigated tissue, we expressed relative *pmel* expression in relation to relative *slc24a5* expression. We found a higher expression level of *pmel* in the posterior than the anterior stripe melanophores in *A. christyi*, and a similar but less significant difference in *B. rhoadesii* (Figs. 1J–1L). No expression gradient was detected in the straight-striped species. Since elevated *pmel* expression is associated with increased pigmentation of melanophores in the stickleback (Greenwood, Cech & Peichel, 2012), the expression gradient of *pmel* may in fact balance the gradient in melanophore density, which is indicated by *slc24a5* expression, in order to result in an evenly colored stripe. The anterior-posterior expression difference of *pmel* in the oblique-striped species may be a result of more clonally distant melanophore populations in Am versus Pm compared to those in the corresponding regions of the straight-striped *M. auratus*.

### Expression patterns of candidate target genes

**Figure 2:**
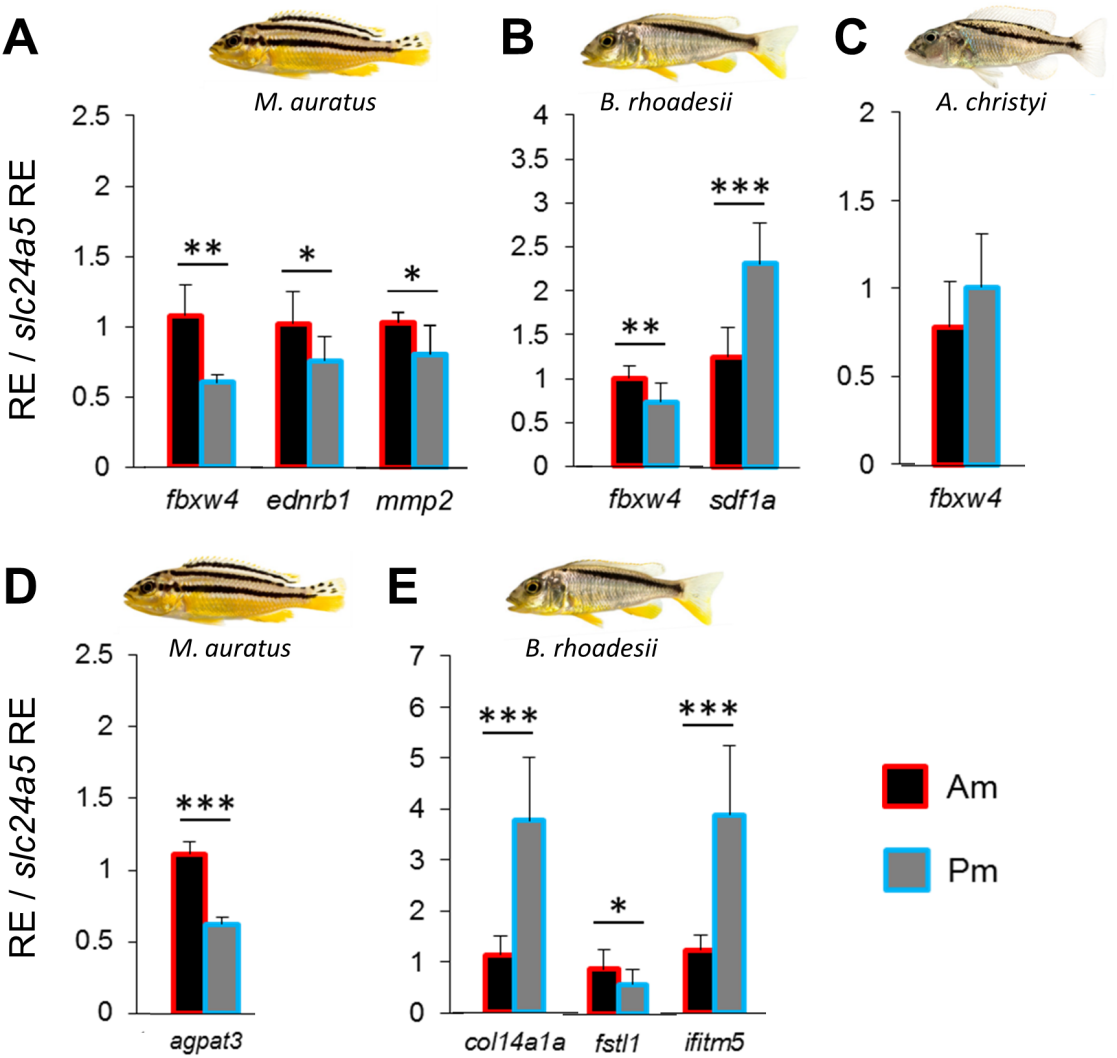
Expression differences of stripe pattern candidate genes along the dark mid-lateral stripe. Bars show the expression levels of primary candidate target genes (A–C) and the co-expressed candidate genes (D, E) with significant anterior-posterior expression differences in the dark stripe region. The relative expression levels in each region are divided by the relative expression of the melanophore marker gene *slc24a5* in that region in order to control for variation in melanophore numbers. The statistical differences are indicated by one, two and three asterisks above bars indicating *P* < 0.05, 0.01 and 0.001, respectively. Error bars represent standard deviations calculated from five biological replicates and RE indicates relative expression.

We compared the expression levels of 11 candidate target genes between anterior and posterior skin samples along the dark mid-lateral stripe (Fig. S1). The selected target genes are involved in adult pigmentation and/or stripe formation, and variation in the expression levels of some of these genes could be a result of melanophore density rather than reflect melanophore properties. To capture expression patterns independent of variation in melanophore density (Ahi & Sefc, 2017), we divided relative target gene expression by the relative expression level of the melanosome marker *slc24a5* (RQ data with and without correction for melanophore density are shown in Table S4). None of the candidate genes showed anterior versus posterior expression differences along the oblique stripe of *A. christyi* (Fig. S1)*,* whereas in the other oblique-striped species, *B. rhoadesii*, expression differences were detected for *sdf1a* (Pm > Am) and *fbxw4* (Am > Pm) (Figs. 2B, 2C). Higher anterior expression of *fbxw4* was also detected in the straight-striped *M. auratus*, along with higher anterior expression of *ednrb1* and *mmp2* (Fig. 2A).

### Expression analyses of co-expressed candidate genes

We extended the primary candidate target gene set by step-wise screening of co-expression networks (see Ahi et al., 2015) using a vertebrate database, COXPRESdb (Obayashi & Kinoshita, 2011). In the first step, we identified the eight most strongly co-expressed genes for two of the differentially expressed primary target genes *sdf1a* and *fbxw4* in *B. rhoadesii* and *M. auratus*, respectively. We used co-expression data from zebrafish for *fbxw4*, and since such data was not available for *sdf1a*, we selected the top ranked genes showing conserved co-expression with the *sdf1a* homologue across chicken, mouse and human (Table S2). Among the new candidate genes co-expressed with *sdf1a* and *fbxw4* only *col14a1a* (Pm > Am) and *fstl1* (Am > Pm) in *B. rhoadesii*, and *agpat3* (Am > Pm) in *M. auratus* showed differential expression (Figs. 2D, 2E and Fig. S2). For each of the two species, there was at least one gene showing strong anterior-posterior differential expression in the same direction as its co-expressed gene, that is, *fbxw4*-*agpat3* and *sdf1a*-*col14a1a*. In the next step, we tested the expression levels of top ranked genes co-expressed in zebrafish with both *fbxw4* and *agpat3*, and with *col14a1a* (no zebrafish data for *sdf1a*). This led to the identification of one additional differentially expressed gene (*ifitm5*; co-expressed with *col14a1a*) in *B. rhoadesii* (Fig. 2E and Fig. S2). The congruent direction of the anterior-posterior expression differences hinted at a gene co-expression module consisting of *sdf1a*-*col14a1a-ifitm5* in *B. rhoadesii*. All of the genes that showed differential expression in one species (i.e., *agpat3*, *col14a1a*, *fstl1* and *ifitm5*) were also tested in the other two species, but none of the expression differences were shared between species (Table S4).

### Expression analyses of predicted upstream regulators

We searched for potential upstream regulators of the identified *sdf1a*-*col14a1a-ifitm5* module in *B. rhoadesii* by predicting potential TF biding sites in the upstream promoter sequences of the genes in the module, based on the high quality annotated genome of the Nile tilapia (Flicek et al., 2013). By implementing two commonly used algorithms for motif enrichment, we identified several motifs enriched in the promoter sequences of the three genes in the module. We parsed the motifs against the known TF binding sites in vertebrates and compiled lists of top potential TFs binding to each motif (Table S3). Finally, we analyzed the expression of 10 most significantly predicted TFs of the module in *B. rhoadesii*, but detected no significant differences in anterior-posterior gene expression levels (Fig. S3).

### Co-expression network and predicted upstream regulators of *pmel*

**Figure 3:**
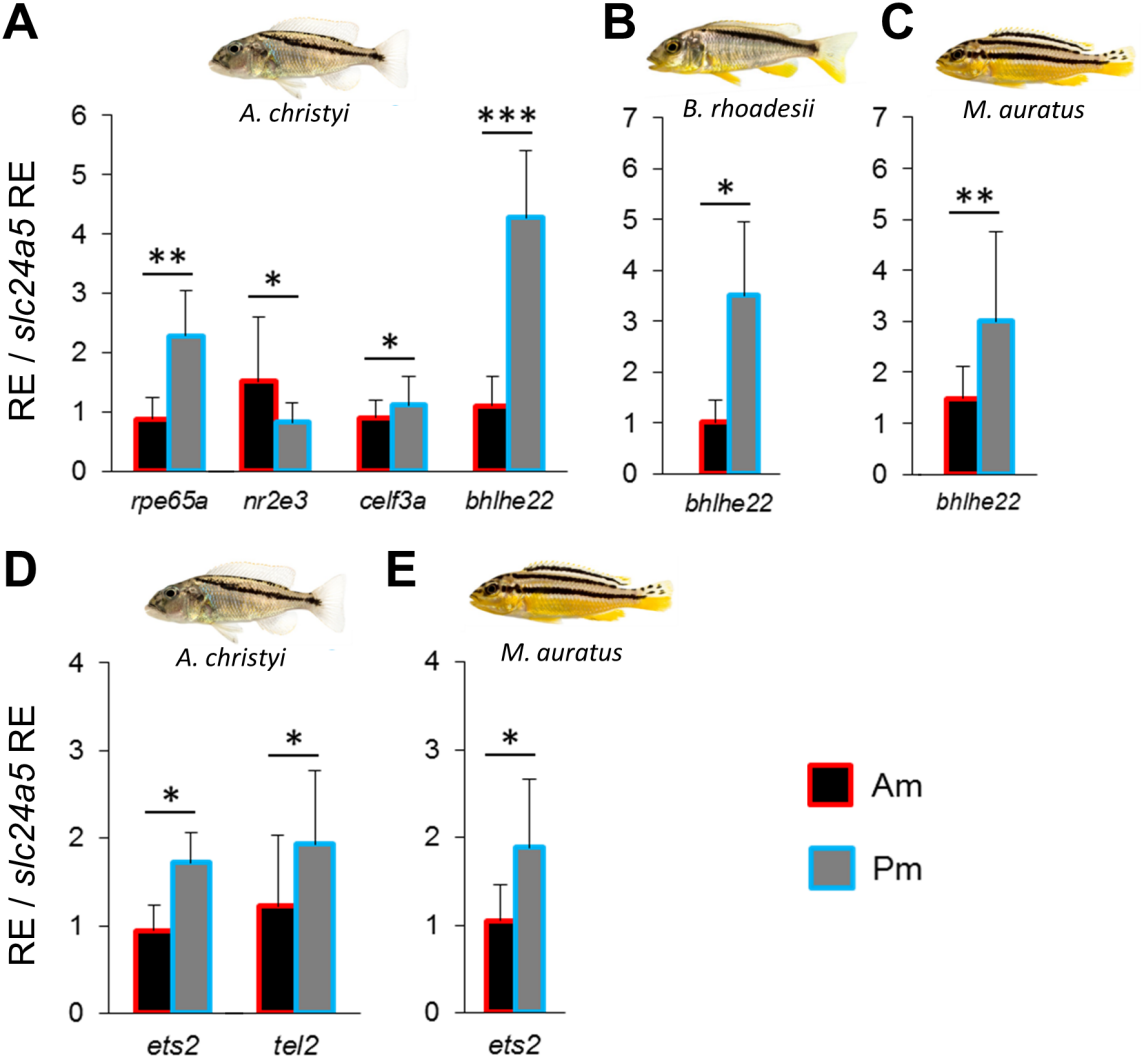
Expression differences of *pmel* related network genes along the dark mid-lateral stripe. Bars show the expression levels of *pmel* co-expressed candidate genes (A–C), and the predicted TFs (D, E), with significant anterior-posterior expression differences in the dark stripe region. The relative expression levels in each region are divided by the relative expression of the melanophore marker gene *slc24a5* in that region in order to control for variation in melanophore numbers. The statistical differences are indicated by one, two and three asterisks above bars indicating *P* < 0.05, 0.01 and 0.001, respectively. Error bars represent standard deviations calculated from five biological replicates and RE indicates relative expression.

In order to identify a co-expression network potentially involved in melanin localization and melanophore shape determination, we repeated the above steps using zebrafish co-expression data for *pmel*. The strong expression gradient of *pmel* in *A. christyi* (Figs. 1J–1L) was mirrored by only one of the tested co-expressed genes, *rpe65a* (Fig. 3A and Fig. S2). In the next step, testing genes co-expressed with both *pmel* and *rpe65a*, differential expression was detected for *nr2e3*, *celf3a* and *bhlhe22* (Fig. 3A and Fig. S2). Since only *rpe65a* and *bhlhe22* showed a strong expression difference in the same direction as *pmel* (Pm > Am), we suggest a gene co-expression module consisting of *pmel*-*rpe65a-bhlhe22* in *A. christyi*. When we tested the genes that were differentially expressed in *A. christyi* in the other two species*,* we found the expression gradient of *bhlhe22* to be shared across the three species (Figs. 3A–3C).

Next, we applied the method described above to predict and examine potential TFs regulating the *pmel*-*rpe65a-bhlhe22* module in *A. christyi*. We detected differential expression of two predicted TFs, *ets2* and *tel2*, in the same direction as the corresponding module genes (Pm > Am) in *A. christyi*, consistent with a transcriptional regulatory role upstream of *pmel*-*rpe65a-bhlhe22* (Fig. 3D). The two differentially expressed TFs identified in *A. christyi* were also tested in the other two species and *ets2* showed higher posterior expression in *M. auratus* but not in *B. rhoadesii* (Fig. 3E)*.*

## Discussion

In this study, we compared potential components of stripe formation mechanisms between cichlid species displaying two distinct stripe patterns, i.e., straight horizontal stripes versus oblique stripes extending at an angle across the length of the fishes’ bodies, in order to identify factors that potentially affect stripe orientation. Studies in the zebrafish model system have shown that melanophore stripe formation depends on the behavior of melanophore populations, which includes migration, differentiation and pigmentation of cells and is influenced by the effects of numerous gene products as well as by interactions with other chromatophores in the integument (Patterson & Parichy, 2013; Frohnhöfer et al., 2013; Mahalwar et al., 2014; Singh & Nüsslein-Volhard, 2015). For instance, it has been shown that melanophores disperse throughout the skin in the absence of other chromatophores (Takahashi & Kondo, 2008), and a model of chromatophore interactions suggests that iridophores repel melanophores on a very short range but cause them to aggregate in their neighborhood, which contributes to the light-dark stripe pattern in the adult zebrafish (Frohnhöfer et al., 2013). Also, melanophores disperse to their nearby space in the absence of iridophores (Patterson & Parichy, 2013), and on the other hand, an expanding high number of iridophores can lead to morphological changes and disappearance of very closely located melanophores (Nüsslein-Volhard & Singh, 2017). In our study, we quantified the expression of iridophore and melanophore marker genes as proxies for the distributions of these chromatophores in the dark and light colored tissue samples. We found increased expression of the iridophore marker gene *ltk* in the anterior tissue samples both around and within the stripe of *A. christyi* and *B. rhoadesii* (Fig. 1)*.* This may indicate a link between an uneven distribution of iridophores along the anterior-posterior axis and the oblique orientation of the stripes in these species. In the straight-striped species, an anterior-posterior difference in *ltk* expression was detected only ventral of the dark stripe. The early emergence of iridophores along the horizontal myoseptum plays a crucial role in orienting the stripes along the anterior-posterior axis (Nüsslein-Volhard & Singh, 2017). In zebrafish larvae, the first emerging iridophores along this axis, as they spread to occupy the available space, serve as morphological pre-pattern for the stripes to form. Subsequently, melanoblasts migrate to the presumptive stripe region, differentiate to melanophores and form dark stripes while interacting with their adjacent iridophores (Frohnhöfer et al., 2013; Nüsslein-Volhard & Singh, 2017). Therefore, the stripe pre-patterning in zebrafish is tightly mediated by proliferation, dispersal and patterned aggregation of iridophores (Singh, Schach & Nüsslein-Volhard, 2014). The stripe patterns observed in the adult females of the three investigated cichlids are retained from their earlier developmental stages. Thus, the uneven distribution of iridophores along the anterior-posterior axis in *A. christyi* and *B. rhoadesii* might be linked to pre-patterning of their oblique melanophores stripe. In other words, the higher number of iridophores in the anterior part might be due to their higher proliferation in this region which can lead to their faster and further dorso-ventral expansion from the myoseptum, and hence, pre-patterning the stripe region further towards dorsal (Singh, Schach & Nüsslein-Volhard, 2014). Further investigations at cellular level, perhaps using iridophore ablation and transplantation approaches, are required to confirm such a link.

The anterior-posterior gradient in the expression of the melanophore marker *slc24a5* suggested a gradient in melanophore numbers along the stripes of *A. christyi* and *B. rhoadesii*, although no variation in stripe coloration was apparent to the naked eye. In order to assess whether the pigmentation of the melanophores varied along the stripe, we investigated the expression levels of *pmel*, a gene determining melanophore structural properties such as cell shape and melanin localization, and possibly melanophore position in skin (Schonthaler et al., 2005). Increased *pmel* expression is associated with dark coloration of melanophores in freshwater threespine sticklebacks (Greenwood, Cech & Peichel, 2012) and in unpaired fins of an East African cichlid fish (Ahi & Sefc, 2017). In order to correct for heterogeneous melanophore densities, which would per se cause variation in *pmel* expression along the stripes, we divided relative *pmel* expression by the relative expression of the melanosome marker gene. This approach follows results of a previous study, where we determined a high correlation between measured *slc24a5* expression and counts of melanophores in the fins of another cichlid fish (*r* = 0.89, *p* < 0.0001; Ahi & Sefc, 2017).

We found a positive anterior-posterior expression of *pmel* in the oblique-striped species, which could potentially contribute to the homogeneous coloration of the stripe in the face of the opposing gradient in melanophore density. In the straight-striped species, which showed no variation in melanophore marker gene expression along the stripe, *pmel* expression along the stripe was invariant as well.

While the association of *pmel* with stripe pigmentation has been shown in various fish (Schonthaler et al., 2005; Greenwood, Cech & Peichel, 2012; Ahi & Sefc, 2017), it is not clear whether *pmel* plays role in melanophore mobility. However, since the transcriptional regulation of *pmel* is not well investigated, it would be interesting to identify transcriptional regulatory mechanism(s) underlying *pmel* anterior-posterior expression difference along the stripe. Analyses of expression patterns of co-expressed genes and predicted transcription factors identified a gene module, *pmel*-*rpe65a-bhlhe22*, and its potential TFs *ets2* and *tel2*/*etv7* in *A. christyi* (Fig. 3). *rpe65a* is required for retinoid metabolism in the visual cycle (Redmond et al., 2005), and is found to be highly expressed in zebrafish melanophores as well (Higdon, Mitra & Johnson, 2013). In mammals, *rpe65a* has been shown to be involved in a process of pigment accumulation in skin (Fan et al., 2006). *bhlhe22* (or *bhlhb5*), encoding a basic helix-loop-helix transcription factor, had higher expression in the posterior stripe regions in all three species, and may therefore have a conserved function in anterior-posterior patterning. In the mouse embryo, *bhlhe22* participates in the development of the retina and the nervous system, with higher expression in the posterior body compartment (Brunelli, Innocenzi & Cossu, 2003; Feng et al., 2006). A mouse lacking *bhlhe22* showed skin lesions mainly in its posterior body parts (Ross et al., 2010). Both *ets2* and *tel2* belong to the ETS family of transcription factors and were found to be expressed in adult zebrafish skin (Quintana et al., 2014; Lisse, King & Rieger, 2016). Furthermore, *tel2* is highly expressed in human epidermal melanocytes (Haltaufderhyde & Oancea, 2014), and *ets2* has been demonstrated to determine the anterior-posterior body axis in mammals and xenopus (Kawachi, Masuyama & Nishida, 2003; Georgiades & Rossant, 2006). When we analyzed both TFs in the other two species, we only found differential expression of *ets2* (Am < Pm; i.e., gradient in the same direction as *tel2*) in the straight-striped *M. auratus*. Interestingly, TEL2 can suppress ETS2 transcriptional activity and this mechanism appeared to be highly conserved in animal (between *Drosophila* and human) (Vivekanand et al., 2012). Consequently, in *A. christyi*, the anterior-posterior variation in *tel2* expression might cancel out the variation in *ets2* expression, thus making the *tel2*/*ets2* expression patterns in the two oblique-striped species functionally equivalent. This also implies that the anterior-posterior expression difference of *pmel* requires absence of ets2 transcriptional/functional difference along this axis.

Among the primary set of candidate genes for stripe orientation, *sdf1a* and *fbxw4* showed the strongest differential expression between the anterior and posterior stripe regions (Figs. 2A, 2B). The *fbxw4*/*hagomoro* gene encodes an F-box/WD40-repeat (or Hag) protein, which is essential for melanophore organization during the formation of the adult pigment pattern of zebrafish. Interestingly, a mutant phenotype of *fbxw4* showed aberrations of the stripe pattern mainly in the anterior trunk region (Kawakami et al., 2000). In East African cichlid fishes, patterns of DNA base substitution and mRNA splicing variation suggest a role of *fbxw4*/*hagomoro* in the diversification of pigmentation patterns (Terai et al., 2002; Terai et al., 2003). Our study detected increased *fbxw4* expression in the anterior stripe regions of *M. auratus* (straight-striped) and *B. rhoadesii* (oblique), but not in the oblique-striped *A. christyi*. Since *fbxw4* is required for the anterior stripe patterning in zebrafish, homogeneous anterior-posterior *fbxw4* expression might be linked to the oblique stripe orientation in *A. christyi*, but apparently not in *B. rhoadesii*, where a gradient in *fbxw4* expression was detected. The qPCR primers used in our study span exons 8 and 9 of *fbxw4* and our mRNA quantification therefore comprised only those splicing variants which contain these two exons (Terai et al., 2003). Consequently, the qPCR result may also indicate differences in splicing, and variation in *fbxw4* isoform composition may exist among species and influence stripe orientation.

The second candidate gene with an expression gradient (Am < Pm) in the oblique-striped *B. rhoadesii*, *sdf1a*, encodes a melanophore-attracting chemokine, which controls migration of melanophores and is required for lateral stripe patterning in zebrafish (Svetic et al., 2007). Expression of *sdf1a* in cells adjacent to the horizontal myoseptum in zebrafish embryos constrains melanophore invasion towards a specific route during their dorsoventral migration and leads to the formation of horizontal stripe (Svetic et al., 2007). However, it is not clear whether *sdf1a* plays the same role during adult pigmentation as well. Variation in *sdf1a* expression along the oblique stripe of adult *B. rhoadesii* may point to a role of *sdf1a* in adult pattern maintenance.

We also identified a slight increase in the expression of *ednr1b*, encoding a receptor of the endothelin pathway, in the anterior stripe region of *M. auratus*. The receptor is expressed in melanophores of embryos and adult zebrafish, but it is only required for adult pigmentation and stripe formation (Parichy et al., 2000). Although *ednrb1* was found to be more crucial for the formation of ventral stripes, its potential role in pigmentation along the anterior-posterior axis remained unexplored. The last gene, *mmp2*, showed slightly increased expression in the anterior stripe region only in *M. auratus*. The gene encodes an extracellular matrix remodelling enzyme which contributes to melanophore migration and body stripe formation in *Xenopus* and zebrafish (Tomlinson et al., 2009; Ellis & Crawford, 2016).

We screened vertebrate co-expression data (mainly from zebrafish) to extend our candidate gene set, and found strong anterior-posterior expression differences in some of the tested genes (Figs. 2D, 2E). The role of these genes in body stripe formation in fish has not been studied and would be a promising subject for future experimental investigations. It is known, however, that *col14a1a* is involved in molecular mechanisms that can be indirectly linked to body stripe patterning. *col14a1a* (Pm > Am expression in *B. rhoadesii*) encodes a member of the collagen family which plays a structural role in dermal-epithelial basement membrane formation during zebrafish embryogenesis (Bader et al., 2013). In adult zebrafish, *col14a1a* is expressed mainly in dermis (Li et al., 2011). Differential *col14a1a* expression between the anterior and posterior stripe regions in *B. rhoadesii* might cause structural changes in dermal layers along this axis, and subsequently affect migration and arrangement of melanophores.

Finally, none of the predicted and tested upstream regulators of the identified module genes *sdf1a*-*col14a1a-ifitm5* in *B. rhoadesii* showed expression differences along the anterior-posterior axis.

## Conclusions

In summary, anterior-posterior expression differences were found along the oblique stripes of *A. christyi* and *B. rhoadesii* as well as along the straight stripe of *M. auratus*. Only one of the detected anterior-posterior expression differences was shared between all three species, and the role of this gene (*bhlhe22*) in color patterning is unclear. Both oblique-striped species differed from the straight-striped species in the spatial distribution of inferred iridophore density and *pmel* gene expression. Furthermore, the divergent expression patterns of the transcription factors *tel2* and *ets2* may result in similar patterns of *ets2* TF activity in the two oblique-striped species. The expression patterns of the candidate genes for adult stripe formation were not correlated with stripe orientation, suggesting that the transition from the common, straight-striped pattern to oblique stripes in *A. christyi* and *B. rhoadesii* is possibly controlled differently in the two closely related species.

##  Supplemental Information

10.7717/peerj.4080/supp-1 Figure S1 Relative expression levels of target genes The expression levels of 11 candidate target genes were compared between anterior and posterior regions of the middle black stripe in three Malawi cichlid species. The relative expression levels in each region are divided by the relative expression of the melanophore marker gene *slc24a5* in that region in order to control for variation in melanophore numbers. The statistical differences are indicated by one, two and three asterisks above bars indicating *P* < 0.05, 0.01 and 0.001, respectively. Error bars represent standard deviations calculated from five biological replicates and RE indicates relative expression.

10.7717/peerj.4080/supp-2 Figure S2 Relative expression levels of genes co-expressed with *pmel*, *sdf1a* and *fbxw4* The expression levels of candidate co-expressed genes were compared between anterior and posterior regions of the middle black stripe in three Malawi cichlid species. The relative expression levels in each region are divided by the relative expression of the melanophore marker gene *slc24a5* in that region in order to control for variation in melanophore numbers. The statistical differences are indicated by one, two and three asterisks above bars indicating *P* < 0.05, 0.01 and 0.001, respectively. Error bars represent standard deviations calculated from five biological replicates.

10.7717/peerj.4080/supp-3 Figure S3 Relative expression levels of predicted transcription factors The expression levels of predicted TFs were compared between anterior and posterior regions of the middle black stripe in three Malawi cichlid species. The relative expression levels in each region are divided by the relative expression of melanophore marker gene *slc24a5* in that region in order to control for variation in melanophore numbers. The statistical differences are indicated by one, two and three asterisks above bars indicating *P* < 0.05, 0.01 and 0.001, respectively. Error bars represent standard deviations calculated from five biological replicates.

10.7717/peerj.4080/supp-4 Table S1 qPCR Primer sequences and gene information

10.7717/peerj.4080/supp-5 Table S2 List of coexpressed candidate genes

10.7717/peerj.4080/supp-6 Table S3 List of predicted transcription factor binding sites

10.7717/peerj.4080/supp-7 Table S4 Raw Cq values and results of the statistical tests
