# Supplementary figures and images for: Anterior-posterior gene expression differences in three Lake Malawi cichlid fishes with variation in body stripe orientation
Source: PeerJ. 2017 Nov 17;5:e4080. doi: 10.7717/peerj.4080 (PMC5695249; doi:10.7717/peerj.4080)

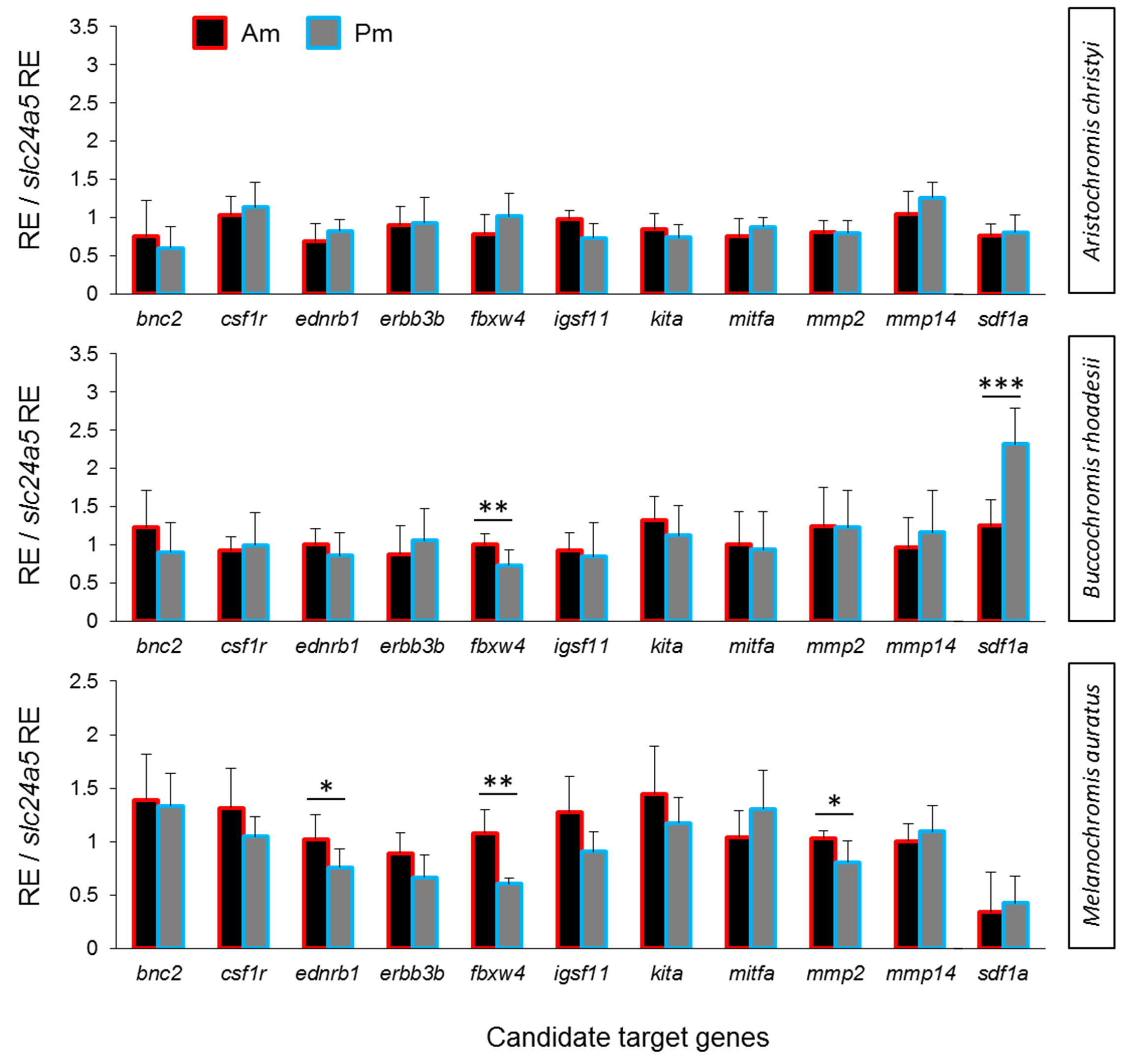

Supplement: Figure S1 — The expression levels of 11 candidate target genes were compared between anterior and posterior regions of the middle black stripe in three Malawi cichlid species. The relative expression levels in each region are divided by the relative expression of the melanophore marker gene slc24a5 in that region in order to control for variation in melanophore numbers. The statistical differences are indicated by one, two and three asterisks above bars indicating P < 0.05, 0.01 and 0.001, respectively. Error bars represent standard deviations calculated from five biological replicates and RE indicates relative expression. [file peerj-05-4080-s001.png]

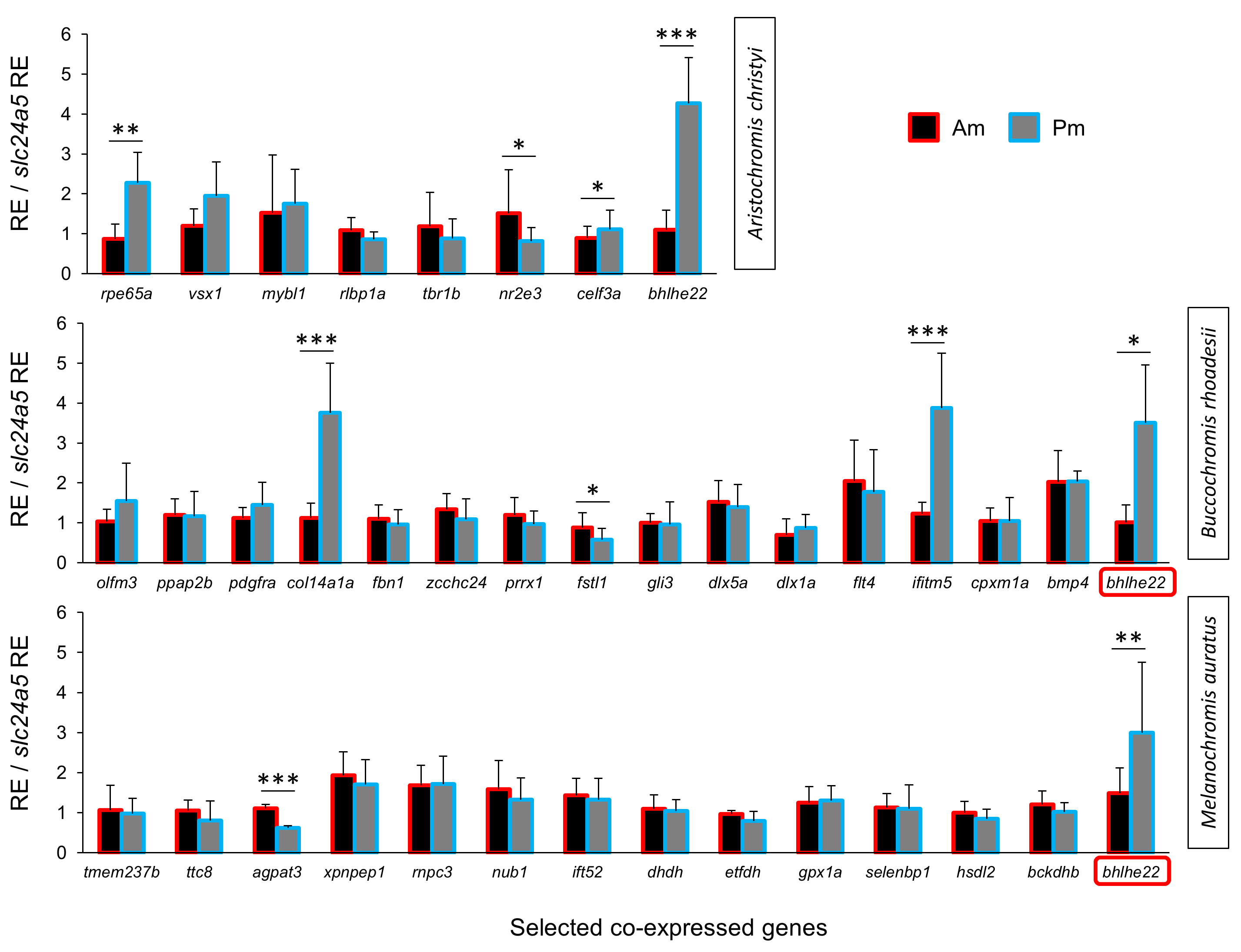

Supplement: Figure S2 — The expression levels of candidate co-expressed genes were compared between anterior and posterior regions of the middle black stripe in three Malawi cichlid species. The relative expression levels in each region are divided by the relative expression of the melanophore marker gene slc24a5 in that region in order to control for variation in melanophore numbers. The statistical differences are indicated by one, two and three asterisks above bars indicating P < 0.05, 0.01 and 0.001, respectively. Error bars represent standard deviations calculated from five biological replicates. [file peerj-05-4080-s002.png]

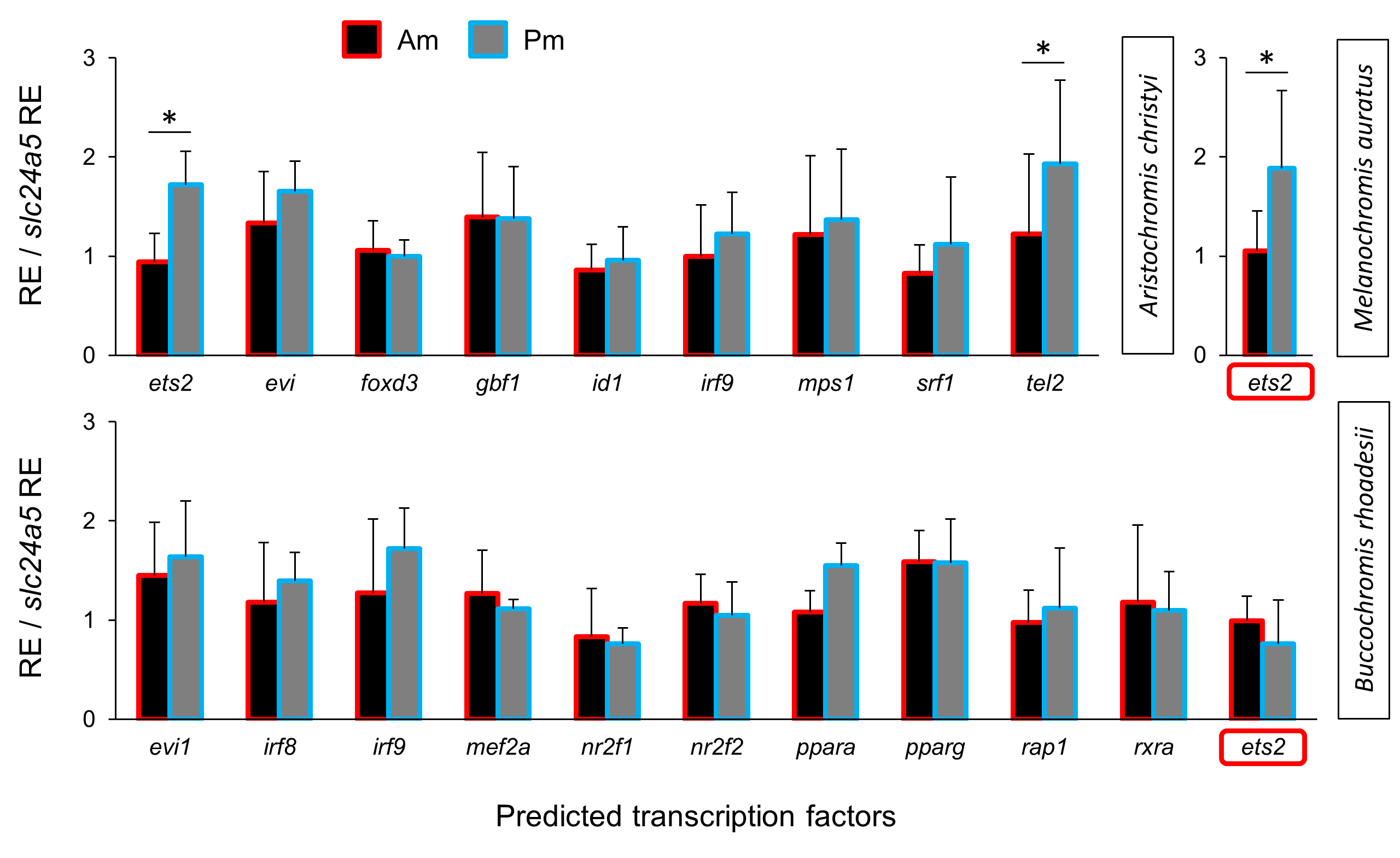

Supplement: Figure S3 — The expression levels of predicted TFs were compared between anterior and posterior regions of the middle black stripe in three Malawi cichlid species. The relative expression levels in each region are divided by the relative expression of melanophore marker gene slc24a5 in that region in order to control for variation in melanophore numbers. The statistical differences are indicated by one, two and three asterisks above bars indicating P < 0.05, 0.01 and 0.001, respectively. Error bars represent standard deviations calculated from five biological replicates. [file peerj-05-4080-s003.png]
